# Buffer 4‐Ethylmorpholinium/Acetate: Exploring a New Alternative Buffer for Native Mass Spectrometry

**DOI:** 10.1002/rcm.10048

**Published:** 2025-04-21

**Authors:** Darya Hadavi, Che Yee Ng, Yuandi Zhao, Anjusha Mathew, Ian G. M. Anthony, Berta Cillero‐Pastor, Eva Cuypers, Tiffany Porta Siegel, Maarten Honing

**Affiliations:** ^1^ Maastricht Multi Modal Molecular Imaging (M4i) Institute, Division of Imaging Mass Spectrometry (IMS) Maastricht University Maastricht The Netherlands; ^2^ MERLN Institute for Technology‐Inspired Regenerative Medicine, Department of Cell Biology‐Inspired Tissue Engineering (cBITE) Maastricht University Maastricht The Netherlands

**Keywords:** charge state distribution, ethylmorpholine, ion mobility spectrometry, native mass spectrometry, protein complexes

## Abstract

**Rationale:**

To perform native mass spectrometry (MS) studies, there are a limited number of volatile and electrospray ionization (ESI)‐MS compatible solutions, such as ammonium bicarbonate and ammonium acetate (AA). These solutions could induce the unfolding of proteins due to the formation of CO_2_ bubbles or induced acidification during ESI. Hence, it was important to introduce a buffer suitable to preserve the native form of proteins while simulating physiological conditions.

**Methods:**

The 4‐ethylmorpholinium/acetate (4EM/A) buffer was compared to AA for the analysis of proteins and protein complexes with mass ranges from 5 to 103 kDa and isoelectric points (pI) between 3 and 11. The evaluations were conducted by comparing the native‐MS profiles, CCS values, arrival time distributions (ATDs), and proteins bioactivities. The human cardiac troponin complex (cTn complex) and its subunit cardiac troponin T (cTnT) were analyzed as proof of the applicability of this buffer for challenging proteins and protein complexes.

**Results:**

4EM/A led to lower charge states compared to AA, supporting the likelihood of preserving protein folding during nano‐ESI and in a high vacuum environment of MS. Ion mobility measurements revealed that proteins in 4EM/A exhibit a lower degree of conformational variation compared to AA, suggesting enhanced conformational stability and potential retention of natural‐like compactness. Additionally, testing the impact of 4EM/A on bioactivity, lysozyme showed increased biological activity in 4EM/A relative to AA, highlighting the buffer's potential for real‐time assessment of protein interaction kinetics and bioactivity. The 4EM/A buffer enabled native‐MS analysis of cTnT for the first time.

**Conclusion:**

We introduced 4EM/A, with pK_a_ of 7.72/4.76, as a promising buffer for native‐MS studies to maintain protein and protein complex bioactivity and conformational integrity.

## 1 Introduction

One of the key factors in unraveling biological processes is to understand the bioactivity of macromolecules, particularly proteins, as a function of their quaternary structures and topology [1]. Techniques such as x‐ray crystallography and nuclear magnetic resonance (NMR) spectroscopy enable high‐resolution studies of protein structures [2]. However, the application of these techniques becomes a challenge when analyzing large, heterogeneous protein complexes or proteins at picomolar concentration levels [3]. Native mass spectrometry (native‐MS) has received growing attention to circumvent these challenges and enable the identification and characterization of pure or mixed proteins with high sensitivity, selectivity, and speed of detection [4]. Native‐MS provides information on the structure, stoichiometry, and molecule–molecule interactions of proteins [5]. However, one of the primary concerns regarding native‐MS studies is the extent to which a charged protein in the gas‐phase retains its native state.

This concern persists, although evidence has demonstrated that native‐MS analysis can preserve the noncovalent interactions and the three‐dimensional (3D) configurations of proteins and complexes [6, 7, 8]. The preservation of a protein's 3D folding is inferred from the characteristics of the ion species produced during native‐MS analysis, which exhibit higher mass‐to‐charge ratio (*m/z*), lower charge states, and narrower charge state distribution compared to those observed in denatured‐MS [9]. To examine whether the bioactivity of proteins is also preserved, Ouyang et al. modified a quadrupole mass spectrometer to enable the soft landing of a selected charged protein on a surface, after traversing through the gas phase of mass spectrometry (MS) [10]. Their results demonstrated that the examined enzymatic re‐solubilized proteins (i.e., trypsin and lysozyme) retained their bioactivity even after electrospray ionization (ESI) and passing through a vacuum. Other studies that performed soft‐landing of protein complexes also evidenced that noncovalent complexes remain intact and the native shape of protein assemblies was retained [11, 12]. Hence, it is safe to state that biomolecules analyzed by native‐MS are at least partially in their native‐like state.

Ion mobility spectrometry (IMS) is a complementary approach to evaluating the native status of ionic proteins. It provides insights into the 3D conformation and folding state of proteins, synthetic polymers [13], and complexes thereof [14]. One method for such analysis is to derive rotationally averaged ion‐neutral collision cross section (CCS) values from IMS measurements [15]. The CCS value offers insights into the tertiary and quaternary structures of protein complexes when analyzing a series of different conditions and charge states [16, 17].

Nano‐ESI has been the most used ionization technique for native‐MS studies. Compared to microflow‐ESI, nano‐ESI provides a uniform response factor, lower sample consumption, and reduced levels of salt added to protein ions [18]. The unified definition of native‐MS, by Leney and Heck, also relies on nano‐ESI for ionization. It defines “native” as the biological state of a macromolecule in the solution just before ionization [19]. This definition emphasizes on careful selection of a solution with appropriate pH and ionic strength to minimize the structural perturbation of proteins while traveling from bulk solution into the fine, charged droplets of nano‐ESI.

To mimic the intracellular environment, biomolecules should be dissolved in non‐denaturing aqueous solvents at neutral pH [20]. To perform native‐MS‐IMS analysis, there are a limited number of volatile and ESI‐MS‐compatible solutions, such as ammonium bicarbonate and ammonium acetate (AA) [21]. Even though the pK_a_ of the HCO_3_^−^ group in ammonium bicarbonate makes it a suitable buffer for native‐ESI‐MS studies, the formation of CO_2_ bubbles within the nano‐ESI droplets could induce the unfolding of proteins [22]. AA has been widely used for native‐MS protein studies at a pH of 6.5–7.0, but this solution does not have a buffer capacity at neutral pH. The lack of buffer capacity is of concern because during positive ionization mode, the nano‐ESI process induces acidification within the ESI capillary [23] and during the shrinkage of droplets in the ESI plume [24]. Protein activity, folding, and charge are pH‐dependent factors [25]. Hence, using a solution without buffer capacity around the physiological pH can induce structural changes in proteins due to pH fluctuations.

To address these limitations in native‐MS studies, we employed 4‐ethylmorpholinium/acetate (4EM/A) buffer for the analysis of proteins and protein complexes with mass ranges from 5 to 103 kDa and isoelectric points (pI) between 3 and 11 (Table S1). The evaluations were conducted by comparing the native‐MS profiles, CCS values, arrival time distributions (ATDs), and bioactivities between 4EM/A and AA. To validate the suitability of the 4EM/A buffer for native‐MS studies of challenging proteins and protein complexes, the human cardiac troponin complex (cTn complex) and its subunit cardiac troponin T (cTnT) were analyzed as the “ultimate” test.

## 2 Material and Methods

### 2.1 Chemicals

Insulin (5.8 kDa, pI 3.32), ubiquitin (8.6 kDa, pI 6.79), RNase (13.5 kDa, pI 8.65), lysozyme (14.38 kDa, pI 10.7), myoglobin from equine heart (17.6 kDa, pI 6.9), tetrameric concanavalin A from *Canavalia ensiformis* complex (monomer 25.5 kDa, pI 4.5–5.5), ammonium acetate (AA), cesium iodide, calcium chloride (CaCl_2_), 4‐ethylmorpholine (or N‐ethylmorpholine (4EM)), phosphate‐buffered saline (PBS) and polyalanine were purchased from Sigma‐Aldrich (Zwijndrecht, The Netherlands). Human cardiac troponin T (cTnT, ~35 kDa, pI 5.13), human cardiac troponin I (cTnI, ~24 kDa, pI 9.87), and cardiac troponin complex (cTn complex, ~77 kDa, C‐subunit pI: 4.02) were purchased from HyTest Ltd. (Turku, Finland). The HPLC‐grade solvents isopropanol (IPA), water (H_2_O), and acetic acid (AcOH) of purity > 98% were purchased from Biosolve BV (Valkenswaard, The Netherlands). The lysozyme activity assay kit (product number ab211113) and RNase activity assay kit (product number ab273299) were purchased from Abcam (Cambridge, UK) and used for the bioactivity test of lysozyme and RNase.

### 2.2 Sample Preparation

The AA solution and 4EM/acetate (4EM/A) buffer (molecule structures in Table S1) were freshly prepared at a concentration of 50 and 200 mM and adjusted to a pH of 7.0. Acetic acid (< 1%) was used for the pH adjustment of 4EM, making up 4EM/acetate, a buffer at a pH range of 7.2–8.2/4.3–5.3. Standard proteins and protein complexes (insulin, ubiquitin, RNase, lysozyme myoglobin, and concanavalin A) were directly dissolved in AA and 4EM/A and diluted to 1 μM for MS studies. Where filtration was needed, the ultracentrifugal filter tube (Amicon Ultra‐0.5 3 kDa or 10 kDa device, Millipore, Darmstadt) made from regenerated cellulose membrane was used. The filtration was performed as instructed in the user guide of the Amicon Ultra‐0.5 by spinning 500 μL of sample at 14000 g for 20 min and recovering the sample by placing the device upside down in a clean microcentrifuge tube and spinning for 2 min at 1000 g using Hettich universal 30 RF centrifuge (Benelux). This solvent exchange process during filtration was performed 3 times consecutively per sample. Concanavalin A was only analyzed after filtration.

To study cardiac proteins, the buffer was first optimized for cTnT and cTnI by comparing their MS profiles in 200 mM of 4EM/A, AA, 4EM/A + IPA (8.3%, pH 7.4), and AA + IPA (8.3%, pH 7.4). IPA was used to increase the solubility of these proteins. Following the selection of 4EM/A + IPA (200 mM, 8.3%, pH 7.4) as the optimized buffer, the cardiac troponin complex, cTnT, and cTnI proteins were filtered and prepared at concentrations of 4.06, 9.46, and 12.57 μM, respectively.

### 2.3 Activity Assay

The activity of lysozyme and RNase proteins was investigated and compared among 4 buffer solutions, namely, a specific protein buffer (from kits), PBS, AA, and 4EM/A (at a concentration of 200 mM and pH 7.0). Lysozyme activity was assessed by following the Abcam ab211113 kit protocol. In short, 4‐methylumbelliferone (4‐MU) and lyophilized lysozyme powder diluted in lysozyme buffer from the kit, PBS, AA, and 4EM/A were adjusted to the final concentrations of 50 pM and 10 mg/mL, respectively. Samples were protected from light and stored on ice for further use. For the activity test, 4‐UM (as a standard), lysozyme protein (as a blank), solvents and lysozyme substrate mix (80%–20%, as blanks), and lysozyme and its substrate (80%–20%, as a test sample) were incubated in a Corning 96‐well plate at 37°C for 1 and 24 h. After the incubation times, a lysozyme stop buffer (1:1) was used to quench the reaction. Released fluorophore, as the consequence of substrate cleavage, was immediately quantified at excitation–emission (Ex/Em) = 360/445 nm and 37°C by the fluorescent microplate reader (Tecan, Switzerland).

The activity of RNase was assessed by following the ab273299 kit protocol from the Abcam kit. Shortly, the investigated solutions (RNase buffer, PBS, AA, and 4EM/A) were first incubated at 65°C for 3 h to reduce RNase contamination. To eliminate the preexisting RNase in the working environment and air, the biosafety cabinet was exposed to 4 h of UV irradiation using the built‐in UV light. The fluorescence standard and lyophilized RNase powder were diluted in RNase buffer from the kit, PBS, AA, and 4EM/A to the final concentrations of 25 pM and 10 mg/mL, respectively. The standard, RNase protein (as a blank), solvents and RNA probe mix (83.3%–16.6%, as blanks), and RNase and RNA probe mix (83.3–16.6%, as a test sample) were added in a black well plate. Sample collection was done every 5 min for 2 h at 25°C, and the released fluorophore was quantified at Ex/Em = 495/520 nm by the fluorescent microplate reader. All activity assays were performed in triplicates.

### 2.4 MS and IMS

The MS measurements were performed using an ultra‐high mass range (UHMR) Q‐Exactive Orbitrap mass spectrometer (Thermo Fisher Scientific, Germany), and IMS‐MS measurements were performed on a Synapt G2‐Si HDMS (Waters, Milford, MA, USA) with traveling wave IMS and quadrupole‐time‐of‐flight mass analyzer (TWIMS‐Q‐TOF). Concanavalin A, cTn complex, cTnT, and cTnI were only analyzed by UHMR Q‐Executive Orbitrap. Pulled, gold‐coated borosilicate glass capillaries (produced in‐house as described before) were used for all experiments with the UHMR Q‐Exactive Orbitrap via a static nanoESI source. Nanospray needles were homemade from preheated borosilicate glass capillaries (Science Products GmbH, Hofheim, Germany) on a DMZ universal electrode puller (Zeitz‐Instruments Vertriebs GmbH, Munich, Germany), followed by gold coating with a SC7640 sputter coater (Quorum Technologies, Kent, UK). For nano‐ESI ionization on TWIMS‐Q‐TOF, the silicon chip‐based nano‐ESI system NanoMate (Advion BioServices, Ithaca, NY, USA) was interfaced to Synapt G2‐Si. All experiments were performed in positive ion mode. The nano‐ESI, MS, and IMS conditions on both instruments are listed in Table S2. Mass calibrations on TWIMS‐Q‐TOF and UHMR Q‐Exactive Orbitrap were performed with cesium iodide, and IMS calibration was done by polyalanine. All MS and IMS measurements were performed in triplicate on 3 consecutive days, and IMS data were reported under the latest recommendations [26].

### 2.5 Data Analysis

The MS data from TWIMS‐Q‐TOF were analyzed with MassLynx v4.1. Arrival time distribution data were analyzed with DriftScope v2.8 software, and mobilities of an ion of interest were extracted and plotted in Excel for further analysis. The MS data from UHMR Q‐Exactive Orbitrap were analyzed by the Thermo Scientific Xcalibur software package. The charge states of peaks in the MS spectra and the mass of the proteins were calculated using ESIprot Online [27]. The UniDec software [28] was used for the deconvolution of the mass spectra. For MS and IMS analysis, 20 scans were averaged. A Python (cPython Version 3.9.7, Python Software Foundation, Wilmington, DE, USA) script using the MassLynx Raw library (Waters) was used to determine the centroids and distribution widths of IMS profiles using formulas from Sivalingam et al. [29] Data were processed with GraphPad Prism 5.

## 3 Results and Discussion

The performance of a 4EM/A buffer for the native‐MS‐IMS studies was compared to that of an AA solution. It was hypothesized that 4EM with a pK_a_ of 7.72 [30] provides a buffering capacity around the physiological pH (7.0), enabling the 4EM/A buffer to outperform AA. To evaluate this hypothesis, four thermodynamically and structurally stable proteins (insulin, ubiquitin, RNase, and lysozyme) and a protein complex (concanavalin A) representing a broad range of mass and pI values were systematically investigated (Table S1).

To confirm that 4EM/A buffer preserves protein activity, the biological activity of lysozyme and RNase was examined. The potential of 4EM/A buffer for native‐MS studies was eventually verified by analyzing the cTn complex and its subunits. Given the significant variation in the polarity of its subunits, the cTn complex is considered a highly challenging target for native‐MS analysis.

### 3.1 Nano‐ESI‐MS

**FIGURE 1:**
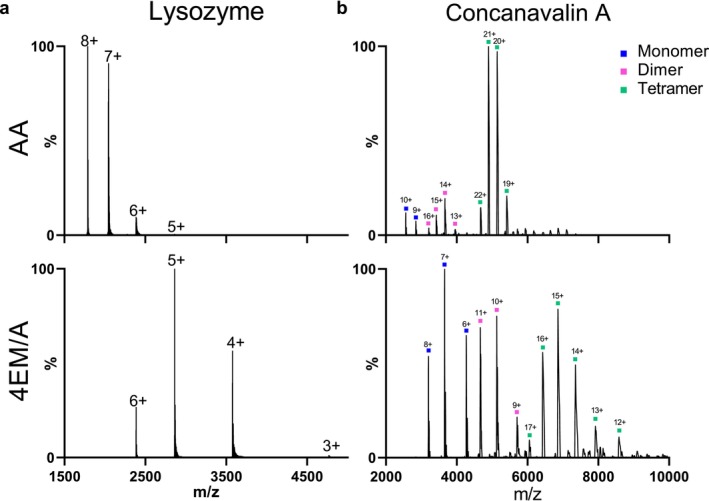
The nano‐ESI‐MS profile of (a) lysozyme protein and (b) concanavalin A protein complex in 200 mM AA and 4EM/A at pH 7.0 analysed by UHMR Q‐Exactive orbitrap.

For both 4EM/A and AA, native‐MS spectra were obtained, yet with different charge state distributions (Figure 1 and Figures S1–S4). Dissolved proteins in 4EM/A buffer, regardless of their pI and molecular mass (MM), tended to form ions with lower charges than proteins in the AA solution. For instance, the charge state distribution of lysozyme in AA ranges from 5^+^ to 8^+^, whereas in 4EM/A, it exhibits a charge state as low as 3^+^ (Figure 1a). In addition, a clear shift of base peaks to the ions with a lower charge state was seen for all proteins in 4EM/A. This trend was also observed for the concanavalin A complex (Figure 1b). The monomer, dimer, and tetramer of concanavalin A were detectable in both solutions. However, the ionized concanavalin A in 4EM/A exhibited up to seven lower charges compared to AA.

The ionic strength of 200 mM was used to prepare proteins in AA and 4EM/A at pH 7.0. The applied ionic strength falls within the range found in both the extracellular and intracellular environments (150–200 mM) [31]. The reported CCS Database for native‐like proteins by both Bush and Wysocki labs is also based on experiments in 200 mM of AA at pH 7.0 [32, 33, 34]. More importantly, such a high ionic strength is essential for maintaining proteins in their native state, as is previously shown for myoglobin in varying concentrations of AA [35]. Nevertheless, to ensure that the charge reduction by 4EM/A is concentration independent, the native‐MS of myoglobin was compared between 50 and 200 mM of the 4EM/A buffer. As shown in Figure S5, 4EM/A enabled myoglobin ionization with a lower charge state than AA, regardless of its concentration.

Samples were analyzed using an UHMR Q‐Exactive Orbitrap as well as a Q‐TOF mass spectrometer equipped with TWIMS. Ionization was carried out using glass capillary‐based nano‐ESI on the Orbitrap and chip‐based nano‐ESI on the Q‐TOF. The nano‐ESI, MS, and IMS conditions on both instruments are listed in Table S2. The impact of 4EM/A buffer was consistent across different mass analyzers and ionization sources (Figures S1–S4).

Overall, these results indicate that the 4EM/A buffer, independent of its concentration, similarly affects both proteins and protein complexes, resulting in native‐MS profiles with lower charge states than AA. However, the main shortcoming of 4EM/A buffer was related to the signal intensity of proteins, which was usually twofold lower than AA. The vapor pressure of 4EM and AA with boiling points of 138.5°C and 117.1°C are 10.85 and 18.53 hPa at 25°C, respectively [36]. Even though both are categorized as volatile solutions, 4EM/A has a lower vapor pressure than AA at a given temperature, reducing its volatility relative to AA [30]. This physical property might explain the lower signal intensity of proteins in 4EM/A. The suppression of MS protein signals was also previously reported for *N*‐methylmorpholine [37]. To further gain insight into the folding state, size, shape, and conformational landscape of proteins the ion mobility experiments were performed.

### 3.2 Nano‐ESI‐IMS‐MS

**FIGURE 2:**
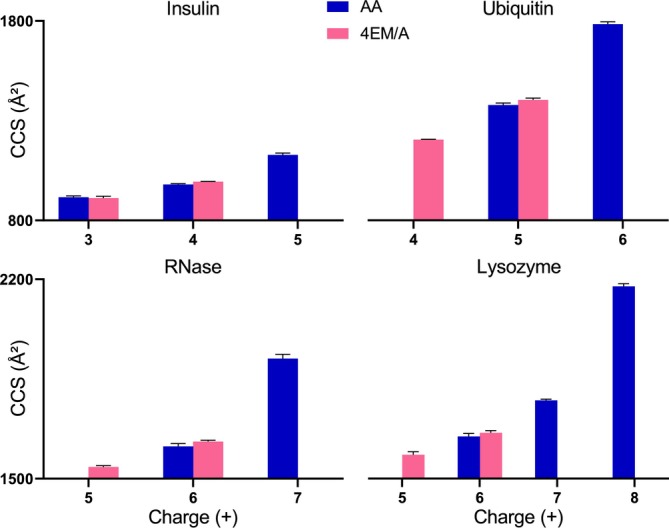
The experimental ^TW^ $\mathrm{CC}S_{N_{2}}$ of four protein standards in 200 mM AA and 4EM/A at pH 7.0 at their observed charge states.

Ion mobility analysis was performed to correlate native‐MS data with the molecular conformations and folding of proteins by deriving ATDs and CCS values and comparing them in between the samples. Table S3 and Figure 2 displays the experimental CCS values, measured by the TWIMS instrument filled with N_2_ (^TW^ $\mathrm{CC}S_{N_{2}}$), for all proteins at their respective observed charge states.

An increase in ^TW^ $\mathrm{CC}S_{N_{2}}$ was observed for all proteins with increasing charge states, indicating a greater degree of unfolding due to the columbic repulsion [38]. In addition, the ^TW^ $\mathrm{CC}S_{N_{2}}$ values showed an unsystematic increase (ranging from 1.15 to1.29 fold) as the charge increased, specifically from 4^+^ to 5^+^ in insulin, 5^+^ to 6^+^ in ubiquitin, 6^+^ to 7^+^ in RNases, and 7^+^ to 8^+^ in lysozyme. Notably, the 1.3‐fold increase between 5^+^ and 6^+^ in ubiquitin was also observed by Stiving et al. [33]. This observation suggests that as protein mass increases, the corresponding rise in ^TW^ $\mathrm{CC}S_{N_{2}}$ is linked to higher charge states. For instance, the notable shift of CCS was observed at 5^+^ in insulin, but for a larger protein like lysozyme, it was observed at a higher charge (8^+^). According to these results, smaller proteins are more sensitive to charge state change. This could be due to the lower surface‐to‐volume ratio of smaller proteins and their limited capacity for compacting. Consistent with these experimental findings, the previously reported molecular dynamics simulations have also shown a higher susceptibility of low mass proteins to charge [39].

Furthermore, Figure 2 shows that low charge state ions, seen only in 4EM/A buffer, have the lowest ^TW^ $\mathrm{CC}S_{N_{2}}$ values. According to the former experimental and simulation results, a small CCS value is usually indicative of a compact and native‐like conformation of a protein [40]. Hence, obtaining the smallest CCS values by 4EM/A highlights the crucial role of this buffer in preserving protein conformations in native‐IMS‐MS studies.

**FIGURE 3:**
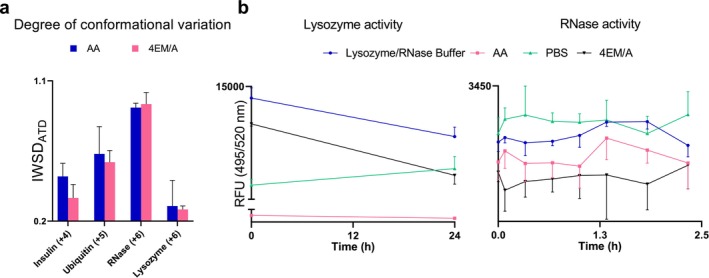
(a) The IWSD_ATD_ of four protein standards at a charge state concurred in AA and 4EM/A. (b) The bioactivity of lysozyme and RNase in different buffers.

When the ^TW^ $\mathrm{CC}S_{N_{2}}$ values of a particular charge state were compared among different solutions, they had comparable values (Figure 2). However, the CCS values cannot be representative of ATD peak width, inflection points (i.e., peaks, shoulders, and tails), and minor conformational changes. To account for these changes, the intensity‐weighted standard deviation of arrival times (IWSD_ATD_) was calculated. The IWSD_ATD_ value, introduced by Thalassinos lab [29], calculates the degree of conformational variation that is acquired in an ATD. Table S4 and Figure 3a shows the IWSD_ATD_ of insulin, ubiquitin, RNase, and lysozyme at the charge states of 4^+^, 5^+^, 6^+^, and 6^+^, respectively, as these charges concurred in both AA and 4EM/A. For three proteins, larger IWSD_ATD_ values and greater variability in the error bars were found in AA compared to 4EM/A, which could be due to a larger variation in their molecular conformation and some degree of instability. In contrast, the ATDs of proteins in 4EM/A were narrower and showed less conformational heterogeneity, which could indicate the contribution of 4EM/A in conformational stability.

The low and narrow charge state distribution observed in MS offers valuable insights into a protein's 3D folding. However, MS alone cannot fully assess the protein's capacity for compaction, nor does it reveal how charge variations correlate with conformation or what degree of conformational alterations occur at a specific charge state. This highlights the importance of complementary IMS analysis, which allows for monitoring CCS value, ATD, and particularly IWSD_ATD_ to gain a more comprehensive understanding of protein structure and dynamics.

### 3.3 Impact of Buffer and Solution on Protein Compactness

The observed differences between 4EM/A buffer and AA solution may be related to their differing salting‐out effects. Ions with strong salting‐out capabilities compete with solute (protein) counter ions for interaction with solvent (water) molecules. This stronger interaction of salt ions with the solvent reduces the solvent–solute (water–protein) interactions, enhancing protein stability and preventing denaturation [41, 42]. Because 4EM/A and AA both possess the same acetate anion, the different behaviour of proteins should pertain to the positively charged cations.

According to the Hofmeister series [43], the ammonium ions, which are classified as salting‐out agents, better preserve the secondary and tertiary structure of proteins [44]. When the ion–protein interaction of the weakly hydrated ammonium ions is considered, ammonium tends to have a weak affinity to the protein backbone and negatively charged side chains, causing salting‐out [44]. This could explain the observed native‐MS behaviors in AA.

When it comes to 4EM ion, it is important to locate it in the Hofmeister series. According to the experimental and simulation studies of Okur et al. [44], cations follow the standard ordering of the Hofmeister series, with the weakly hydrated cations interacting less strongly with proteins and causing salting‐out. Because the 4EM ion is larger than ammonium, it is likely less hydrated than ammonium and hence interacts more weakly with proteins. This weaker interaction could explain why proteins are less unfolded in 4EM/A compared to AA.

In addition, both AA and 4EM/A were prepared at a rather high concentration of 200 mM, similar to the ionic strength found in extracellular and intracellular environment [31], which is pivotal for maintaining proteins in their native state [35]. The salting‐out effect is even more pronounced by increasing the ionic strength of an aqueous sample, which subsequently reduces the ionization degree of proteins [45]. It is because of the increased interaction between the solvent and added salt ions, which eventually reduces the availability of water molecules to interact with proteins to form hydrogen bonds.

Besides, one of the key factors to maintain the structural stability of proteins is a steady pH. It is especially important throughout the nano‐ESI process that includes acidification [46]. Unlike AA, 4EM/A (pK_a_ 7.72/4.76) has a buffer capacity around the physiological pH, which could build resistance against pH change and consequently enable proteins to preserve their native conformation. Kramer et al. [47] also introduced 4EM to perform better than ammonium for metal interaction studies, as it did not interfere with copper binding to peptides and proteins. Studies on 2,2‐difluoroethylamine and 2,2,2‐trifluoroethylamine, which have buffer capacity at physiological pH, also indicated to be better replacements to AA for native‐MS studies [48]. However, they showed similar charge state distribution as AA.

The higher salting‐out capability of 4EM, particularly at elevated ionic strength, combined with its buffering capacity at physiological pH, could explain the greater degree of protein compactness observed in 4EM/A compared to AA. To confirm that the observed compactness does not alter the biological function of proteins, an activity assay was performed on the enzymatic protein's lysozyme and RNase.

### 3.4 Activity Assay

Figure 3b (left) shows the bioactivity of lysozyme in lysozyme buffer from the kit, PBS, AA, and 4EM/A after 1 and 24 h of incubation. After an hour of incubation, the maximum activity of lysozyme was observed in lysozyme buffer followed by 4EM/A buffer. In contrast, this protein demonstrated the poorest activity in the AA solution. Even though the bioactivity of lysozyme protein in 4EM/A buffer was reduced over 24 h of incubation, still its activity was comparable to PBS, the commonly used buffer in biological research. The reduced activity of lysozyme in 4EM/A buffer might be related to the autoxidation of residual Fe^2+^ in 4EM/A buffer, as it has been evident for 3‐morpholinopropane‐1‐sulfonic acid (MOPS) buffer [49]. This phenomenon could change the ion composition of the buffer and influence the 3D conformation of the protein and consequently its function. However, further ion composition analysis is required for confirmation. The RNase protein was active right after the RNA probe introduction (Figure 3b, right). The reaction of RNase and its probe fitted well with the Michaelis–Menten model. Unlike lysozyme, the highest activity of RNase was observed in the PBS buffer and the least activity was seen in the 4EM/A buffer. Interestingly, the results of the RNase activity assay could be related to the findings from IMS inspections, where IWSD_ATD_ values from 4EM/A were greater than AA (unlike other proteins). These IMS results indicate that RNase was experiencing some degree of instability and conformational variation in 4EM/A, which could directly affect its bioactivity. Nevertheless, this protein was still biologically active in 4EM/A. Therefore, it is evident that 4EM/A is a potential buffer for native‐MS‐IMS studies, because of its properties in preserving the 3D conformation and biological function of proteins. However, the slight degree of change in the presence of 4EM/A appeared to be protein dependent.

### 3.5 Native‐MS of Cardiac Troponin Complex and Its Subunits

The cTn complex is of special clinical importance due to its significant role in cardiac muscle activity and heart failure. This complex consists of three subunits, namely, cTnT, cTnI, and troponin C (TnC). Considering the significance of this complex in early diagnosis, it is of value to elucidate its complete structure, conformation, and interaction of the subunits with each other. However, to the best of our knowledge, there is no native‐MS study on the complex or even the cTnT subunit. Previous MS studies on cardiac troponin proteins were mainly performed in the denatured form of these proteins and the complex [50, 51]. To fully understand the interaction of subunits and relate the quality of these interactions to cardiovascular diseases, it is essential to characterize the cardiac troponin complex and its subunits in their native form. Hence, the applicability of 4EM/A buffer for the native‐MS study of the cTn complex and its subunits (cTnT and cTnI) was examined.

**FIGURE 4:**
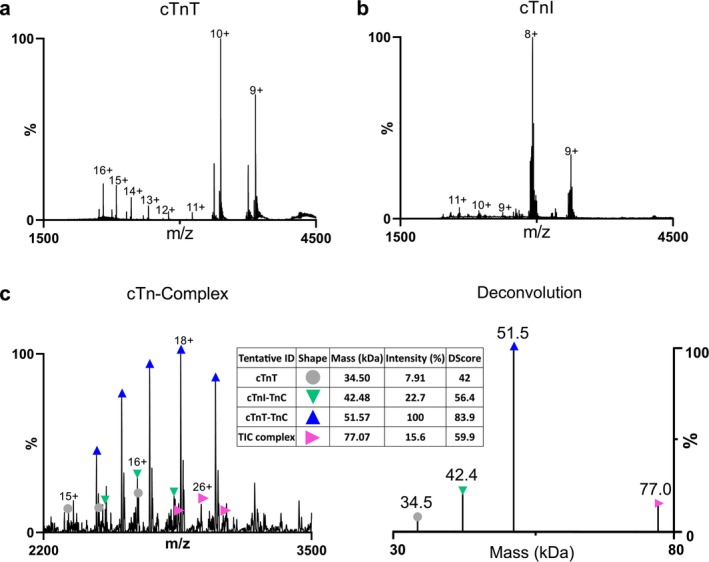
The nano‐ESI‐MS profile of (a) cTnT and (b) cTnI in 200 mM 4EM/A + IPA solution (8.3%, pH 7.4). (c) The native‐ and deconvoluted MS of the cTn complex in 4EM/A + IPA.

Before analysing the cTn complex, first, the buffer was optimized for detecting cTnT and cTnI individually in 200 mM of 4EM/A, AA, 4EM/A + IPA (8.3%, pH 7.4), and AA + IPA (8.3%, pH 7.4). IPA was used to increase the solubility. Although cTnI was easily ionized in the tested buffers, detecting cTnT in its native form was challenging. The native‐MS of cTnT was only obtained using the 4EM/A buffer, with further enhancement by adding IPA. No cTnT was detected in AA with or without varying percentages of IPA. The optimized 4EM/A + IPA buffer (200 mM, 8.3%, pH 7.4) enabled the detection of native cTnT with a base peak at 10^+^ followed by 9^+^ (Figure 4a). The charge state distribution of denatured cTnT between 11^+^ and 16^+^ was also detected at low intensities. This could be because of the presence of the organic solvent in the buffer.

The measured molecular mass of 34.5 kDa (Table S1) corresponds to cTnT isoform 6 lacking the first methionine amino acid on the N‐terminal and N‐acetylated serine 2. In addition, another proteoform of cTnT with the molecular mass of 33.8 kDa was detected, which might pertain to its isoform with 281 amino acids. When 4EM/A + IPA was used to analyze cTnI, the measured molecular mass was 23.6 kDa, corresponding to the monophosphorylated form of cTnI isoform 3 (Figure 4b) [50].

As the optimized buffer of 4EM/A + IPA (200 mM, 8.3%, pH 7.4) preserved the compact form of both subunits in the gas phase, the same buffer composition was used to analyze the cTn complex. Figure 4c shows the native‐MS and deconvoluted MS of the cTn complex, with a peak distribution attributing to the expected molecular mass of 77 kDa. However, the charge state distribution deviates from a typical native‐MS profile. The low intensity and tendency of the complex to dissociate into its subunits with minor changes in ionization conditions indicated its low stability and fragile nature.

The molecular masses at 34.5 and 42.4 kDa likely correspond to the cTnT and the cTnI‐TnC binary complex, respectively. The most abundant peak at 51.5 kDa could be attributed to the cTnT–TnC binary complex, with one subunit in fragment form. Detecting cTnT as an individual subunit suggests a low affinity of this protein to other subunits in the complex. These observations suggest that the TnC subunit may interact more strongly with both cTnT and cTnI. On the contrary, cTnT and cTnI appear to lack a stable interaction, as no peak for a cTnT‐cTnI binary complex were detected. Although tandem mass spectrometry (MS/MS) was applied to fragment the tertiary and binary complexes for identification, the signal‐to‐noise ratio of the spectra was too low for conclusive interpretation. Further experiments are required to confirm these peaks, which is beyond the scope of this paper. Overall, the 4EM/A buffer not only enabled the first successful detection of native‐MS for cTnT but also facilitated the detection of tertiary and binary complexes of cardiac proteins.

After evaluating the total of 7 proteins (insulin, ubiquitin, RNase, lysozyme, myoglobin, cTnI, and cTnT) and 2 protein complexes (concanavalin A and cTn complex), it could be concluded that 4EM/A buffer maintains the solution phase folding of proteins and proteins complexes for native‐MS.

## 4 Conclusion

Considering the significance of native‐MS studies in understanding the properties and behavior of proteins spatially and kinetically, it is important to address challenges in preserving the native form of proteins while simulating physiological conditions. We introduced 4EM/A, with pK_a_ of 7.72/4.76, as a promising buffer for native‐MS studies to maintain protein and protein complex conformational integrity. Compared to an AA solution, the 4EM/A buffer resulted in lower charge states for all examined proteins and complexes. Consequently, proteins with lower CCS values attributing to the higher degree of compactness and potentially preserved natural folding state were achieved by 4EM/A. In addition, comparing ATDs demonstrated that 4EM/A buffer contributes to greater conformational stability than AA. The preservation of enzymatic protein bioactivity (lysozyme and RNase) in 4EM/A further supports the buffer's ability to maintain native‐like structures. These findings highlight the benefits of using 4EM/A in conformational studies, enzyme kinetics, real‐time analysis, and binding assays. This paper also emphasizes the need for complementary IMS investigations, including CCS values, ATDs, and IWSD_ATD_. Furthermore, 4EM/A enabled the first detection and analysis of cardiac troponin complex and its subunit, cardiac troponin T, potentially providing new insights into their role in cardiac events.

## Author Contributions

**Darya Hadavi:** conceptualization, investigation, writing – original draft, methodology, validation, visualization, writing – review and editing, software, formal analysis, project administration, data curation. **Che Yee Ng:** methodology, data curation. **Yuandi Zhao:** methodology, data curation. **Anjusha Mathew:** methodology. **Ian G. M. Anthony:** software. **Berta Cillero‐Pastor:** funding acquisition, writing – review and editing. **Eva Cuypers:** writing – review and editing. **Tiffany Porta Siegel:** writing – review and editing. **Maarten Honing:** conceptualization, funding acquisition, investigation, writing – review and editing, supervision, resources, project administration.

## Supporting information


**Table S1.** List of the examined proteins and protein complexes, their respective isoelectric point, and molecular mass obtained from nano‐ESI‐UHMR Q‐Exactive Orbitrap and TWIMS‐Q‐TOF.
**Table S2.** The nano‐ESI, MS, and IMS conditions on UHMR Q‐Exactive Orbitrap and TWIMS‐Q‐TOF.
**Table S3.** The experimental ^TW^ $\mathrm{CC}S_{N_{2}}$ of four protein standards in 200 mM AA and 4EM/A at pH 7.0 at their observed charge states.
**Table S4.** The IWSD_ATD_ of four protein standards at a charge state concurred in AA and 4EM/A.
**Figure S1.** MS profile of insulin in 4EM/A and AA by UHMR Q‐Exactive Orbitrap and TWIMS‐Q‐TOF.
**Figure S2.** MS profile of ubiquitin in 4EM/A and AA by UHMR Q‐Exactive Orbitrap and TWIMS‐Q‐TOF.
**Figure S3.** MS profile of RNase in 4EM/A and AA by UHMR Q‐Exactive Orbitrap and TWIMS‐Q‐TOF.
**Figure S4.** MS profile of lysozyme in 4EM/A and AA by UHMR Q‐Exactive Orbitrap and TWIMS‐Q‐TOF.
**Figure S5.** MS profile of myoglobin in 200 mM of AA and 50 and 200 mM of 4EM/A by TWIMS‐Q‐TOF.

## Data Availability

The data that support the findings of this study are available in the supplementary material of this article.
