# Supplementary material for: Buffer 4‐Ethylmorpholinium/Acetate: Exploring a New Alternative Buffer for Native Mass Spectrometry
Source: Rapid Commun Mass Spectrom. 2025 Apr 21;39(14):e10048. doi: 10.1002/rcm.10048 (PMC12010238; doi:10.1002/rcm.10048)
Supplement: Supplementary file 1 — Table S1. List of the examined proteins and protein complexes, their respective isoelectric point, and molecular mass obtained from nano‐ESI‐UHMR Q‐Exactive Orbitrap and TWIMS‐Q‐TOF. Table S2. The nano‐ESI, MS, and IMS conditions on UHMR Q‐Exactive Orbitrap and TWIMS‐Q‐TOF. Table S3. The experimental TW CCSN2 of four protein standards in 200 mM AA and 4EM/A at pH 7.0 at their observed charge states. Table S4. The IWSDATD of four protein standards at a charge state concurred in AA and 4EM/A. Figure S1. MS profile of insulin in 4EM/A and AA by UHMR Q‐Exactive Orbitrap and TWIMS‐Q‐TOF. Figure S2. MS profile of ubiquitin in 4EM/A and AA by UHMR Q‐Exactive Orbitrap and TWIMS‐Q‐TOF. Figure S3. MS profile of RNase in 4EM/A and AA by UHMR Q‐Exactive Orbitrap and TWIMS‐Q‐TOF. Figure S4. MS profile of lysozyme in 4EM/A and AA by UHMR Q‐Exactive Orbitrap and TWIMS‐Q‐TOF. Figure S5. MS profile of myoglobin in 200 mM of AA and 50 and 200 mM of 4EM/A by TWIMS‐Q‐TOF. [file RCM-39-e10048-s001.docx]

**Supporting Information**

**Buffer 4-ethylmorpholinium/acetate: Exploring a new alternative buffer for native mass spectrometry**

**Darya Hadavi^1*^, Che Yee Ng^1^, Yuandi Zhao^1^, Anjusha Mathew^1^, Ian G. M. Anthony^1^, Berta Cillero-Pastor^1,2^, Eva Cuypers^1^, Tiffany Porta Siegel^1^, Maarten Honing^1*^**

**^1^**Maastricht Multi Modal Molecular Imaging (M4i) Institute, Division of Imaging Mass Spectrometry (IMS), Maastricht University, Maastricht, The Netherlands

**^2^**MERLN Institute for Technology-inspired Regenerative Medicine, Department of Cell Biology-Inspired Tissue Engineering (cBITE), Maastricht University, Maastricht, the Netherlands

*Corresponding author: M.Honing@maastrichtuniversity.nl

Table of Contents

[Supplementary Tables 3](#_Toc193624899)

[Supplementary Figures 6](#_Toc193624900)

[Supplementary Reference 11](#_Toc193624901)

# Supplementary Tables

*Table S1. List of the examined proteins and protein complexes, their respective isoelectric point and molecular mass obtained from nano-ESI-UHMR Q-Exactive Orbitrap and TWIMS-Q-TOF.*

| **Protein/**  **Protein Complex** | **Isoelectric point (pI)** | **Theoretical molecular**  **mass (Da)** | **Measured molecular mass (Da)**  **In AA*** | **Measured molecular mass (Da)**  **In 4EM/A*** | **Mass spectrometer** |
| --- | --- | --- | --- | --- | --- |
| **Insulin** | 3.32 | **Monomer:**  5,807 | 5,807.0 | 5,807.4 | TWIMS-Q-TOF |
|  |  | **Dimer:**  11,614 | 11,615.0 | 11,615.0 | TWIMS- Q-TOF |
|  |  | **Monomer:**  5,807 | 5,806.7 | 5,807.5 | UHMR Q-Exactive Orbitrap |
|  |  | **Dimer:**  11,614 | 11621.9 | 11622.0 | UHMR Q-Exactive Orbitrap |
| **Ubiquitin** | 6.79 | 8,565 | 8,564.2 | 8,564.8 | TWIMS- Q-TOF |
|  |  |  | 8,564.6 | 8,572.0 | UHMR Q-Exactive Orbitrap |
| **RNase** | 8.65 | 13,683 | 13,681.7 | 13,682.1 | TWIMS- Q-TOF |
|  |  |  | 13,682.7 | 13682.3 | UHMR Q-Exactive Orbitrap |
| **Lysozyme** | 10.7 | 14,307 | 14,305.1 | 14,304.7 | TWIMS- Q-TOF |
|  |  |  | 14,297.5 | 14,304.8 | UHMR Q-Exactive Orbitrap |
| **Myoglobin** | 6.9 | ~17,600 | 17,576 | 17,574 | TWIMS- Q-TOF |
| **Concanavalin A** | 4.5-5.5 | **Monomer:**  25,539 | 25,610.1 | 25598.2 | UHMR Q-Exactive Orbitrap |
|  |  | **Dimer:**  51,078 | 51,316.8 | 51305.8 |  |
|  |  | **Tetramer:**  102,156 | 102,783.2 | 102,671.5 |  |
| **Cardiact Troponin I (cTnI)** | 9.87 | ~24,000 | - | 23,642.32 | UHMR Q-Exactive Orbitrap |
| **Cardiac Troponin T (cTnT)**  **Isoform 6** | 5.13 | ~35,000 | - | 34,510.3 | UHMR Q-Exactive Orbitrap |
| **Cardiac Troponin Complex (cTn-complex)** | - | ~77,000 | - | 77,070 | UHMR Q-Exactive Orbitrap |
| *AA:  ammonium acetate | 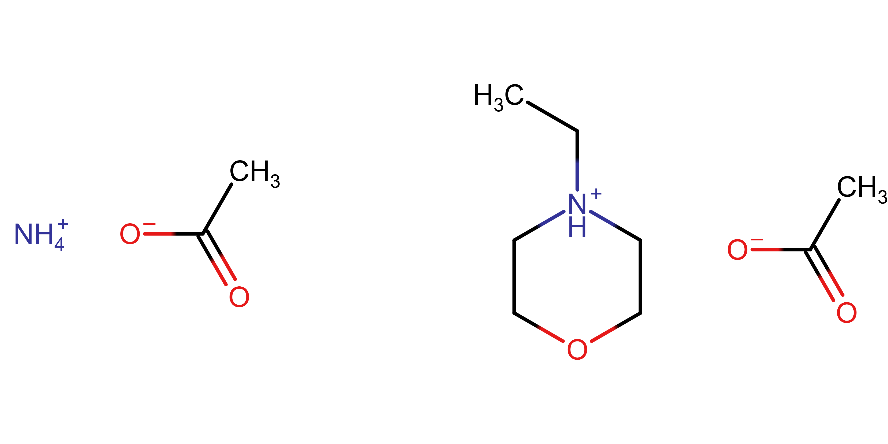 | | *4EM/A:  4-thylmorph  olinium/acetate | 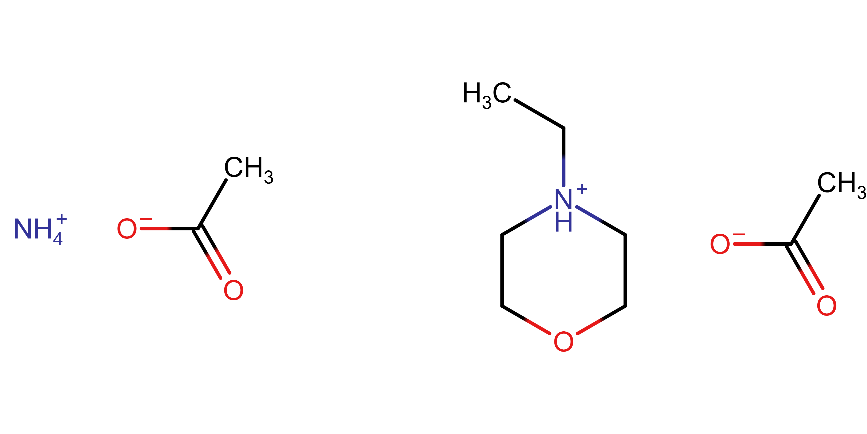 | |

*Table S2. The nano-ESI, MS, and IMS conditions on UHMR Q-Exactive Orbitrap and TWIMS-Q-TOF.*

| **UHMR Q-Exactive Orbitrap** | | **TWIMS-Q-TOF** | | |
| --- | --- | --- | --- | --- |
| **Nano-ESI** | **MS** | **Nano-ESI** | **IMS** | **MS** |
| Spray voltage:  1.1-1.50 kV | Resolution at *m/z* 200  280,000 for proteins  4375 for Concanavalin A | Spray voltage:  1.55 kV  Gas pressure: 0.3 psi | IMS gas rate:  90 mL/min | High-resolution mode |
| S-Lens RF:  200 V | *m/z* range adjusted per analyte | Sampling cone voltage:  120 V | Wave velocity:  600 m/s |  |
| Inlet capillary temperature:  320 °C | Average: 0  Microscans: 1 | Source temperature:  140 °C | Wave height:  40 V | *m/z* range adjusted per analyte |
| Insource trapping voltage:  -50 to -100 V | Injection time:  90 ms | Desolvation gas temperature:  80 °C | Helium gas rate:  180 mL/min |  |

*Table S3. The experimental ^TW^*$CCS_{N_{2}}$ *of four protein standards in 200mM AA and 4EM/A at pH 7.0 at their observed charge states.*

|  | **Charge state** | **^TW^**$\mathbf{CC}\mathbf{S}_{\mathbf{N}_{\mathbf{2}}}$**(Å²)** | |
| --- | --- | --- | --- |
|  |  | **AA** | **4EM/A** |
| **Insulin** | 3 | 916.4 | 912.1 |
|  | 4 | 980.7 | 994.6 |
|  | 5 | 1129.0 | - |
| **Ubiquitin** | 4 | - | 1204.5 |
|  | 5 | 1378.3 | 1403.8 |
|  | 6 | 1783.3 |  |
| **Rnase** | 5 | - | 1540.9 |
|  | 6 | 1613.3 | 1630.1 |
|  | 7 | 1921.2 | - |
| **Lysozyme** | 5 | - | 1583.7 |
|  | 6 | 1647.7 | 1660.7 |
|  | 7 | 1774.2 | - |
|  | 8 | 2174.5 | - |

*Table S4. The IWSD_ATD_ of four protein standards at a charge state concurred in AA and 4EM/A.*

| **Degree of conformational variation** | **IWSD_ATD_** | |
| --- | --- | --- |
|  | **AA** | **4EM/A** |
| **Insulin (+4)** | 0.45 | 0.34 |
|  | 0.36 | 0.34 |
|  | 0.65 | 0.37 |
| **Ubiquitin (+5)** | 0.59 | 0.57 |
|  | 0.65 | 0.54 |
|  | 0.65 | 0.62 |
| **Rnase (+6)** | 0.92 | 0.92 |
|  | 0.98 | 0.96 |
|  | 0.89 | 0.96 |
| **Lysozyme (+6)** | 0.28 | 0.25 |
|  | 0.35 | 0.31 |
|  | 0.26 | 0.26 |

# Supplementary Figures

Figure S1. MS profile of Insulin in 4EM/A and AA by UHMR Q-Exactive Orbitrap and TWIMS-Q-TOF.

Figure S2. MS profile of Ubiquitin in 4EM/A and AA by UHMR Q-Exactive Orbitrap and TWIMS-Q-TOF.

Figure S3. MS profile of RNase in 4EM/A and AA by UHMR Q-Exactive Orbitrap and TWIMS-Q-TOF.

Figure S4. MS profile of Lysozyme in 4EM/A and AA by UHMR Q-Exactive Orbitrap and TWIMS-Q-TOF.

Figure S5. MS profile of Myoglobin in 200 mM of AA and 50 and 200 mM of 4EM/A by TWIMS-Q-TOF.
